# Measuring Airway Inflammation in Asthmatic Children

**DOI:** 10.3389/fped.2018.00196

**Published:** 2018-07-06

**Authors:** Laura Tenero, Marco Zaffanello, Michele Piazza, Giorgio Piacentini

**Affiliations:** Department of Surgical Sciences, Dentistry, Gynecology and Pediatrics, University of Verona, Verona, Italy

**Keywords:** biomarkers, inflammation, FeNO, exhaled breath condensate, exhaled breath volatile compounds

## Abstract

Asthma is the most common chronic respiratory disease in children characterized by airways inflammation, bronchial hyperresponsiveness, recurrent reversible airways obstruction, and respiratory symptoms. The diagnosis of the disease is based on clinical history, airways obstruction at spirometry, and bronchial reversibility. Asthma treatment is aimed to disease control, through the use of controller treatment and monitoring lung function. However, lung function and symptoms not always reflect the underlying airways inflammation and response to the therapy. Objective parameters of asthma inflammation could be important for the clinician in the management of patients with asthma. In the last years, some studies were focused on biomarkers to identify phenotype, inflammation, and pathobiological pathways to help the clinician in the diagnosis and in personalizing the management. Accordingly, clinically feasible tests are represented by the collection of exhaled breath condensate (EBC) and measurement of exhaled nitric oxide (FeNO). Other—methods such as the evaluation of volatile organic compound (VOCs), that reflect airways inflammation and treatment efficacy, are currently used for research purposes For some of these methods, The lack of standardization in pre-collection, collection, post-collection of samples, and interpretation of the results may a problem in clinical practice. Improved these limitations, several biomarkers will be useful to distinguish patients with a different disease condition to personalize the treatment.

## Introduction

Asthma is a chronic respiratory disease characterized by inflammation of the airways, bronchial hyperresponsiveness, recurrent reversible airway obstruction, and respiratory symptoms (1). Asthma is the most common chronic respiratory disease in childhood with an incidence between 1 and 18% in people from different countries.

The diagnosis of asthma is based on anamnestic data, clinical evaluation, limitation of the airflow, and bronchial reactivity (1). The goal of asthma treatment is the control of respiratory symptoms with controller daily drugs, reduction of rescue medications needed to maintain a regular daily activity and reaching a normal lung function (1). Though airways inflammation is a pivotal characteristic of the disease, it is not directly related to lung function test or symptoms (2) and with the real response to the therapy (3).

Therefore, objective parameters of airway inflammation should be considered relevant for the treatment choice in asthmatic patients.

The assessment of biomarkers in the exhaled breath of patients with asthma and other lung diseases is a very attractive approach to monitor airways inflammation. Biomarkers are objectively measurable indicators of the biological and pathological processes as well as of the pharmacological responses to the therapeutic intervention (4).

An ideal biomarker should be easy to collect and measure, inexpensive, noninvasive, and feasible in children capable to contribute to the phenotyping of the disease and to the assessment of treatment response (4, 5).

**Figure 1:**
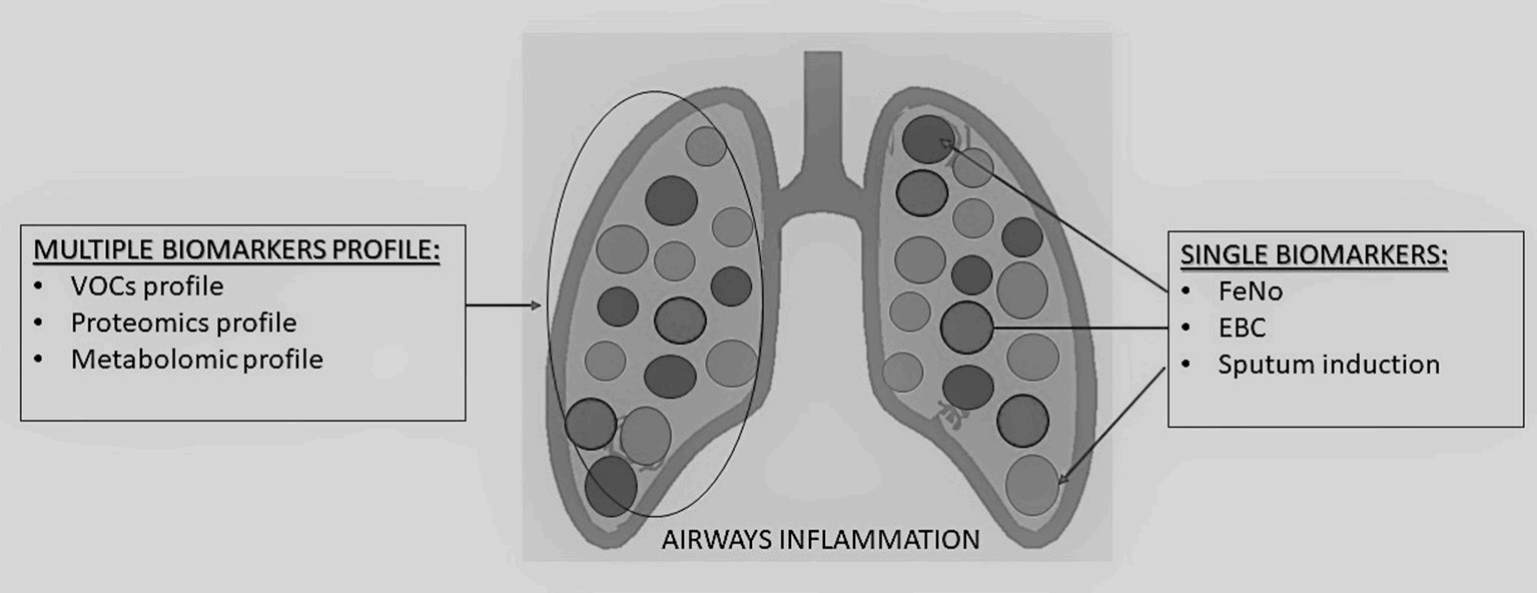
Summary of single and multiple biomarkers detection methods to assess airways inflammation in children.

In order to have a clinically useful tool, there are important issues regarding sensibility, reproducibility, and variability of the methods, that need to be evaluated before moving from bench to bedside. In the next future, a number of recently proposed methods are expected to be clinically useful to predict the progression of the disease, to the phenotyping and endotyping of the disease in order to move toward a personalized treatment in asthma (Figure 1).

Bronchoscopy with bronchoalveolar lavage (BAL) and endobronchial biopsy are considered the gold standards for assessing airway inflammation and remodeling in asthma but, being invasive methods, they have a limited use in the clinical setting, in particular in pediatric care (2).

In recent years, therefore, researchers have focused their studies to define surrogate biomarkers to be proposed in the assessment of airway inflammation in asthmatic children and are shortly revised in the following sections (4–6) (Figure 1).

## Induced sputum

The method of induced sputum is a relatively noninvasive diagnostic procedure able to harvest cells and mediators from the lower airway (2) which, however, requires a level of expertise in the collection in order to obtain adequate samples and reliable results. Sputum can be induced in children older than 6 years after inhalation of nebulized hypertonic saline solution at increasing concentration (7, 8).

The procedure to collect sputum is standardized by European Respiratory Society Task Force and is applicable to children older than 8 years (9, 10).

However, the practical use of this method is limited due to technical issues which can make the feasibility at clinical setting difficult to perform for regular assessment of airways inflammation in children (11, 12). In particular, hypertonic saline inhalation may cause bronchoconstriction, especially in asthmatic children, for whom pre-medication with β2-agonist may be necessary. Furthermore, the post-collection analysis needs skilled laboratory personnel for mediator assay and cell specimens to be transferred onto slides and properly stained. Trained physicians are also needed in order to have a correct reading of the results from the specimens.

Several studies demonstrated a strong correlation between cellular elements of BAL and cells collected from in the airways through the induced sputum method.

The dominant cell in sputum from normal children is the macrophages, and the normal ratio for eosinophils in sputum from children is 2.5% (7).

Different inflammatory patterns are being identified from sputum of asthmatic children and adults*:* eosinophilic, neutrophilic, mixed, and paucigranulocytic types (13).

Sputum eosinophilia is a marker of severity of allergic inflammation in asthma and is characterized by elevated numbers of eosinophils, and eosinophil cationic protein concentration, as well as increased nitric oxide and hydrogen peroxide levels in exhaled breath. The patients with an eosinophilic pattern are expected to have a better response to corticosteroids therapy (14).

Also, children with stable asthma show increased number of eosinophils and bronchial epithelial cells in their sputum (15, 16). During asthma exacerbation, eosinophils and mast cells are more represented in the samples obtained from the airways through the method of induced sputum and eosinophil cationic protein concentrations are higher in the fluid phase of the samples. Some patients have sputum neutrophilia with improved levels of interleukin 8 (17). On the other hand, children with asymptomatic airway hyperresponsiveness are expected to have normal cell counts, whereas patients with cystic fibrosis have sputum neutrophilia (15, 18).

## Exhaled breath condensate

Exhaled breath condensate (EBC) is a noninvasive method aimed to evaluate volatile markers and inflammatory mediators that may contribute to evaluate asthma pathophysiology.

EBC collects particles from airway lining fluid by the condensation of warm humid breath onto a cold surface in a condensing device.

EBC collection, as described in ATS/ERS guidelines, requires a refrigerated device (19) and patient are requested to breath at tidal volume for 10–15 min. During this time the airways lining fluid undergoes an aerosolization process and the exhaled fraction is condensed in a cooling device (0 to −20°C) (20).

The most frequently evaluated parameters in EBC are pH, exhaled markers of oxidative stress and inflammation.

EBC is composed of water vapor, unstable volatiles such as CO_2_ and H_2_O_2_, inorganic (O_2_, N_2_), and organic (CO_2_) particles, exogenous, and endogenous organic compounds, protein, and cytokines (21). In the respiratory tract, H_2_O_2_ may be released from inflamed cells—including neutrophils, macrophages, eosinophils, and epithelial cells. Nitrogen redox forms such as nitrite (NO_2_ –) and nitrate (NO_3_ –) are present in the epithelial lining fluid of the human respiratory tract.

High concentrations of NO_2_ and NO_2_+NO_3_ were showed in patients with asthma, CF and bronchiectasis compared with healthy controls (21).

The pH of EBC is a non-specific marker of airway disease being a median normal pH value of 8.0 in children from 0 to 20 years (22). Some studies showed a lower pH in children with stable asthma than healthy controls and a lower pH value in children with severe than mild asthma (23, 24). In addition, asthmatic patients not adequately treated with ICS have been demonstrated to have a lower pH than those properly treated. Patients with acute exacerbation had a higher pH value after the treatment with budesonide (23, 24).

At present, no association has been reported between asthma symptoms, lung function, FeNO, and airway hyperresponsiveness (25, 26).

Biomarkers related to oxidative stress like H_2_O_2_, 8-isoprostane, asymmetric dimethylarginine (ADMA), aldehydes, and nitrite/nitrate are important to be evaluated in EBC in a number of airway diseases, including asthma.

Asymmetric dimethylarginine (ADMA) is an EBC marker of oxidative stress which can be assessed by the UPLC-MS/MS technique. It is an analog of L-arginine that reduces the synthesis of NO and increases superoxide from inhibition of NOS. A previous study showed that asthmatic children had higher values of ADMA than healthy ones with no difference with ICS treatment (27).

The oxidation of the phospholipid membrane and polyunsaturated fatty acid produces aldehydes and lipid hydroperoxides. One study showed high levels of glutathione in the EBC of asthmatic children with exacerbation and after 5 days of prednisolone therapy, the malondialdehyde level dropped, while glutathione rose (28). Malondialdehyde levels also correlate with air pollution, lung function and inflammatory markers. These results suggest that during exacerbations there is an imbalance between oxidative and antioxidant agents in the airways.

H_2_O_2_ is released from cells in the airways as superoxide anions, an unstable and reactive particle. In the respiratory system, H_2_O_2_ can be released from both inflammatory cells—including neutrophils, macrophages, eosinophils—and epithelial cells. In non-asthmatic, non-smoking children, the normal value of this molecule is 0.09 μmol (19). H_2_O_2_ was higher in asthmatic children during exacerbations and decreased after ICS treatment, supporting the hypothesis that H_2_O_2_ is a marker of airways inflammation (29, 30). However, other studies failed to demonstrate its ability to predict exacerbations (31, 32).

8-isoprostane, a suitable marker of oxidative stress, is a product of arachidonic acid (33). Children and adults with severe asthma or asthma exacerbation have high levels of this 8-isoprostane (34). The concentrations of 8-isoprostane failed to show any correlation with lung function, FeNO, ICS or leukotriene receptor antagonist therapy (35, 36).

Eicosanoids are another group of markers derived from arachidonic acid that play a role in asthmatic inflammation. The presence of these markers in EBC is confirmed by specific enzyme immunoassay and radioimmunoassays (37).

Asthmatic children have high levels of leukotriene B4 (LTB4), cysteinyl leukotrienes (LTC4, LTD4, and LTE4) in EBC (36, 37). The role of cysteinyl leukotrienes (CysLT) in response to ICS therapy is under debate (38–40). Some authors showed a significant reduction of CysLT after a course of oral corticosteroids and after 6 months of ICS therapy, whereas others did not confirm this result. A significant reduction of CysLTs has been reported after montelukast therapy (41).

Several other markers of inflammation and oxidative stress, such as cytokines and adenosine, have been investigated. Asthmatic children showed high Th2 cytokines and low Th1 cytokines in EBC (42, 43). Moreover, children with asthma had a high IL-4/INFγ ratio related to Th2 inflammation (43). IL-4 was high in asthmatic and atopic children as a predictor of asthma condition, whereas IL-5 could predict exacerbations (32).

## Fractional exhaled nitric oxide (FeNO)

Nitric oxide in airways is mainly produced by two enzymes: constitutive nitric oxide synthase (cNOS), that produces low quantities of NO, and epithelial inducible NOS (iNOS) that is induced by various inflammatory cytokines (44). FeNO is a marker of eosinophilic airway inflammation, able to evaluate the level of inflammation and the response of anti-inflammatory therapy (45).

FeNO is a noninvasive, repeatable and reproducible method (46) applicable in the pediatric practice. The gold standard technique for cooperative children is the single breath on-line method (47) but also other techniques have been proposed for uncooperative children or in sedated infants (47). Nevertheless, at present, no clear evidence is available regarding the potential clinical application of FeNO measurements in uncooperative children. In this age group, the method deserves additional efforts to standardization because it its potential application to predict asthma (33).

FeNO levels can be influenced by different factors such as patient's age, height, gender, and race, nasal contamination, exhalation flow ambient and air pollution (48). The execution of spirometry or exercise before the measurement, diet or exposure to smoke also need to be considered (48).

The standardization of techniques permits to collect comparable data from different centers in healthy children and subjects with diseases. For this purpose, the first document on FeNO evaluation in children was published in 2002 (47), subsequently revised by ATS/ERS in 2005 (48).

Several studies demonstrated that FeNO correlates with airway hyperresponsiveness, IgE serum levels, bronchodilator response, skin prick tests, asthma symptoms, and lung function (49, 50).

Airway inflammation in allergic asthma results from the activation of Th2-mediated pro-inflammatory cytokine mechanism involving IL-4, IL-5, and IL-13. This mechanism causes the expression of epithelial inducible NO synthase up-regulated via STAT-6, a process which is corticosteroid sensitive (51, 52).

Furthermore, other studies showed that FeNO levels are correlated with serum eosinophilic cation protein, eosinophils in induced sputum, blood eosinophilia, eosinophil infiltration of the airways, and IgE levels in atopic patients (52). High FeNO values characterize Th2-mediated airway inflammation, eosinophilia, and responsiveness to ICS (52).

FeNO is helpful during evaluation of the patients characterizing asthma of the eosinophilic phenotype and predicting asthma exacerbation. In children less than 5-year-old with recurrent coughing and wheezing, increased FeNO levels can predict physician diagnosed asthma at school age (53). Furthermore, increased FeNO levels at the age of 4 years, predict higher risk for wheezing, asthma and need of ICS by school age (54).

The ATS guidelines recommend the use of FeNO for monitoring airway inflammation and for addressing the choice of anti-inflammatory treatment (52, 55, 56). It has been extensively proved a rapid decrease of FeNO values when ICS treatment is started, with a dose-dependent mechanism and a sudden rise when ICS therapy is withdrawn (57). This trend may be helpful in monitoring patient adherence to the therapy (46).

High FeNO values are not always linked to eosinophilic asthma but also to allergic rhinitis, eosinophilic bronchitis and allergen or viral exposure and a correct interpretation is stressed in the ATS/ERS document (55).

In patient treated with omalizumab, FeNO values, blood eosinophils and BMI can predict the response to the therapy (58).

Authors, for a long time, have been very cautious to support the use of FeNO as coadjutant to standard symptom-based management (59, 60).

However, in recent Cochrane review it has been showed that FeNO guided treatment in asthmatic children was associated with a significant reduction in exacerbation as compared with guideline-based treatment (61). On these bases, the most recent GINA document in 2018 included FeNO guided treatment as a proposed for tailoring treatment to be considered in children (1).

Therefore, FeNO can be regarded a useful method to categorize patients with eosinophilic and Th2-mediated asthma evaluating the response to ICS therapy, predicting exacerbations and the compliance to the therapy.

## Exhaled breath volatile organic compounds (VOCs)

Exhaled breath volatile organic compounds (VOCs) are analyzed by breathomics science and they represent a noninvasive tool to evaluate the lung inflammation. VOCs have a metabolic origin from larger molecules. Airway VOCs originate not only from the upper and lower airways but also from a capillary bed near the alveoli (62). Their concentration in exhaled breath is influenced by blood gas coefficient, cardiac output and alveolar minute volume.

The methodological approach to collect VOCs from exhaled breath requires attention to exclude organic compounds from the ambient air, type of sampling (total vs. alveolar breath), type of collecting materials and other confounding factors (62).

In particular, the collection of airway VOCs needs an inhalation filter to exclude ambient VOCs. The patient is asked to breath into a system which can collect online samples directly via inert tubes into an analyzer or off-line by collecting exhaled air into bags, tubes or syringes. These devices are made of inert materials such as Tedlar bags (63).

Gas chromatography-mass spectrometry (GC-MS) and flame ionization detection (GC-FID) are the most widely used techniques to analyze the samples after collection. These methods can differentiate and quantify VOCs at low concentrations, but they require both qualified technicians and expensive technology (33).

A new non-selective approach to analyze VOCs in exhaled breath is metabolomic profiling that identify and quantify all metabolites in a biological sample without *a priori* hypothesis.

Metabolomic profiles describe the interaction among environmental exposure, medication, nutrition, and toxic substances, genetic expression and microorganisms (33, 62). This method is an interesting approach to patient characterization and personalized medication (33). This approach simultaneously evaluates many metabolites in a sample and generates a profile capable of discriminating between different groups of individuals characterizing the biochemical processes underway in each biological system.

More recently sensor-based device such as the electronic nose, colorimetric sensor array, and gold nanoparticle sensors have been proposed. They adopt specific sensors with optical, chemical or electronic properties that analyze VOCs in the EB (62). Recently, some studies have demonstrated the clinical application of these instruments in respiratory disease (64, 65). VOCs in the EB discriminate between asthma and healthy and between atopic and non-atopic children (64, 65). VOCs profile in exhaled breath was able to discriminate healthy, transient wheezing and asthmatic children starting from the age of 2-3-year-old (66). In the pediatric field, VOCs can also predict asthma exacerbations (67, 68).

Nevertheless, further studies are necessary to evaluate the clinical utility of VOCs in evaluating asthma severity and monitoring asthma symptoms and response to ICS therapy.

## Conclusions

Noninvasive techniques to collect and analyze airways inflammatory biomarkers are helpful in evaluating the airway pathophysiology of asthmatic children.

In clinical practice, FeNO evaluation has been suggested as the only valid and non-invasive technique to test for underlined eosinophilic inflammation, but it would need to be adopted in combination with other useful markers.

The standardization of the new techniques to collect biomarkers in EB and EBC remains problematic. Low reproducibility of exhaled biomarkers and the lack of standardization of the methods of pre-collection, collection, post-collection of the samples, along with the correct interpretation of the results, represent critical issues in clinical practice.

The identification and utilization of ideal and defined biomarkers in asthmatic children remains debated. The reasons for this are the biological aspects of each prospective biomarker, the disease pathobiology, and methods and invasiveness of sample collection. Therefore, the development of novel biomarker with more sensitivity and specificity may lead to prompt diagnosis of severe asthma in future.

## Author contributions

MZ, MP, and LT: drafting of the manuscript; GP, MZ, and LT: critical revision.

### Conflict of interest statement

The authors declare that the research was conducted in the absence of any commercial or financial relationships that could be construed as a potential conflict of interest.
